# Pink1 interacts with *α*-synuclein and abrogates *α*-synuclein-induced neurotoxicity by activating autophagy

**DOI:** 10.1038/cddis.2017.427

**Published:** 2017-09-21

**Authors:** Jia Liu, Xue Wang, Yongquan Lu, Chunli Duan, Ge Gao, Lingling Lu, Hui Yang

**Affiliations:** 1 Center of Parkinson's Disease Beijing Institute for Brain Disorders, Beijing Key Laboratory on Parkinson's Disease, Key Laboratory for Neurodegenerative Disease of the Ministry of Education, Beijing Center of Neural Regeneration and Repair, Department of Neurobiology Capital Medical University, Beijing 100069, China; 2 Capital Medical University affiliated Beijing Anzhen Hospital, Beijing Institute of Heart, Lung and Vessel Disease, The Key Laboratory of Remodeling Related Cardiovascular Disease, Beijing Collaborative Innovation Center for Cardiovascular Disorders, Beijing, 100029, China

## Abstract

Parkinson’s disease (PD) is one of the most common neurodegenerative diseases, characterized by degeneration of dopaminergic neurons in the substantia nigra. *α*-synuclein (*α*-syn) and PTEN-induced putative kinase (PINK)1 are two critical proteins associated with the pathogenesis of PD. *α*-syn induces mitochondrial deficits and apoptosis, PINK1 was found to alleviate *α*-syn-induced toxicity, but the mechanistic details remain obscure. Here, we show that PINK1 interacts with *α*-syn mainly in the cytoplasm, where it initiates autophagy. This interaction was dependent on the kinase activity of PINK1 and was abolished by deletion of the kinase domain or a G309D point mutation, an inactivating mutation in the kinase domain. Interaction between PINK1 and *α*-syn stimulated the removal of excess *α*-syn, which prevented mitochondrial deficits and apoptosis. Our findings provide evidence for a novel mechanism underlying the protective effects of PINK1 against *α*-syn-induced neurodegeneration and highlight a novel therapeutic target for PD treatment.

Parkinson’s disease (PD) is the second most common neurodegenerative disorder and is characterized by motor and non-motor deficits caused by degeneration of dopaminergic neurons in the substantia nigra and formation of intraneuronal proteinaceous inclusions known as Lewy bodies (LBs).^1^

*α*-synuclein (*α*-syn), encoded by *SNCA*, is the main component of LBs; its mutation and overexpression contribute to the pathogenesis of PD.^2^ Mitochondrial dysfunction also plays a major role in the disease.^3^ Many studies have reported that *α*-syn impairs the mitochondrial respiratory chain and thereby reduces mitochondrial membrane potential (MMP), which leads to mitochondria-dependent apoptosis.^4, 5^ Thus, preventing the localization of *α*-syn to mitochondria and mitochondrial quality control are potential strategy for protecting against neurodegeneration in PD.^6, 7, 8^

Phosphatase and tensin homolog deleted on chromosome ten (PTEN) induced putative kinase (PINK)1 is associated with autosomal recessive forms of PD.^9^ PINK1, the 581-amino acid protein, has an N-terminal mitochondrial targeting signal, putative trans-membrane segment, Ser/Thr kinase domain (residues 156–509) and a C-terminal regulatory domain.^10^ Mutations in PINK1 area common cause of early-onset PD;^11^ however, like *α*-syn, PINK1 is absent in LBs of sporadic PD cases.^12^ Some studies showed that PINK1 overexpression suppressed *α*-syn-induced toxicity,^13, 14, 15^ where as its deficiency exacerbated neurodegeneration associated with *α*-syn,^16, 17^ although the underlying mechanisms remain obscure.

Given that PINK1 has a protective role in mitochondrial function, in this study we investigated whether PINK1 can mitigate the cytotoxic effects of *α*-syn. We found that PINK1 interacts with *α*-syn through its kinase domain in the cytoplasm, thereby blocking *α*-syn localization to mitochondria and alleviating *α*-syn-induced toxicity. We also found that this interaction resulted in ubiquitination of the PINK1–*α*-syn complex and consequent degradation of *α*-syn by autophagy.

## Results

### PINK1 protects cells against injury induced by *α*-syn overexpression

**Figure 1:**
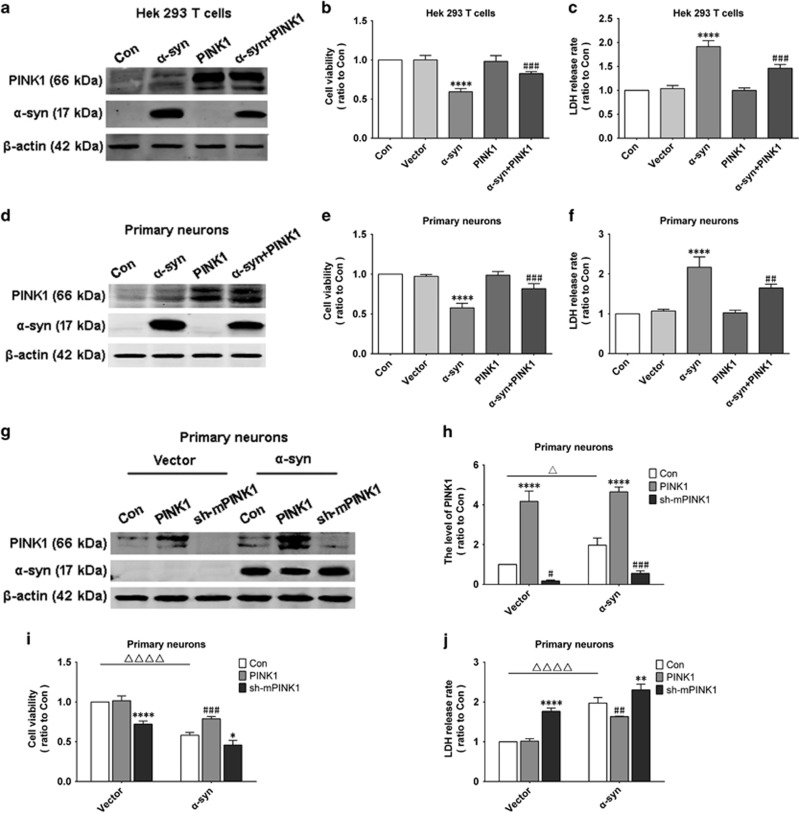
PINK1 alleviates cytotoxicity induced by *α*-syn overexpression. (**a**,**d**). PINK1 and *α*-syn were overexpressed in HEK293T cells and rat primary cortical neurons. (**b**,**c**,**e**,**f**). Cell viability and cytotoxicity were assessed with the MTT and LDH assays, respectively, in HEK293T cells and rat primary cortical neurons. (**g**). PINK1 overexpression or knockdown in C57BL/6mouse primary cortical neurons with or without *α*-syn overexpression. (**h**). Quantification of PINK1 levels. (**i**,**j**). Cell viability and cytotoxicity were evaluated in C57BL/6 mouse cortical primary neurons. Data are expressed as mean±S.D. (**c**–**f**), one-way analysis of variance; (**h**–**j**), two-way analysis of variance. In (**c**–**f**): ^##^*P*<0.01, ^###^*P*<0.001 *versus α*-syn; ****P<0.0001 *versus* control (Con) (*n*=3). In (**h**–**j**): **P<0.01, ***P<0.001, ****P<0.0001 *versus* control (Con); ^#^*P*<0.05, ^##^*P*<0.01, ^###^*P*<0.001, ^####^*P*<0.0001 *versus* control (Con); ^Δ^*P*<0.05, ^ΔΔΔΔ^*P*<0.0001 *versus* control (Con) in the vector group (*n*=3)

Human embryonic kidney (HEK)293T cells or primary neurons from rat cortex were transfected or infected with *α*-syn and PINK1 (Figures 1a and d). To determine whether PINK1 has protective effects in cells overexpressing *α*-syn, cell viability and cytotoxicity were evaluated with the methylthiazolyldiphenyl-tetrazolium bromide (MTT) and lactate dehydrogenase (LDH) assays, respectively. In both HEK293T cells and primary neurons, *α*-syn overexpression decreased cell viability, an effect that was diminished by co-expression of PINK1 (Figures 1b and e). Similar results were obtained with the LDH assay (Figures 1c and f). Knock down of endogenous mPINK1 in primary neurons from mouse cortex (Figures 1g and h) and endogenous PINK1 deficiency (Figures 1i and j) decreased cell viability and increased cytotoxicity, indicating that PINK1 protects cells against injury induced by *α*-syn.

### PINK1 rescues mitochondrial dysfunction induced by *α*-syn

**Figure 2:**
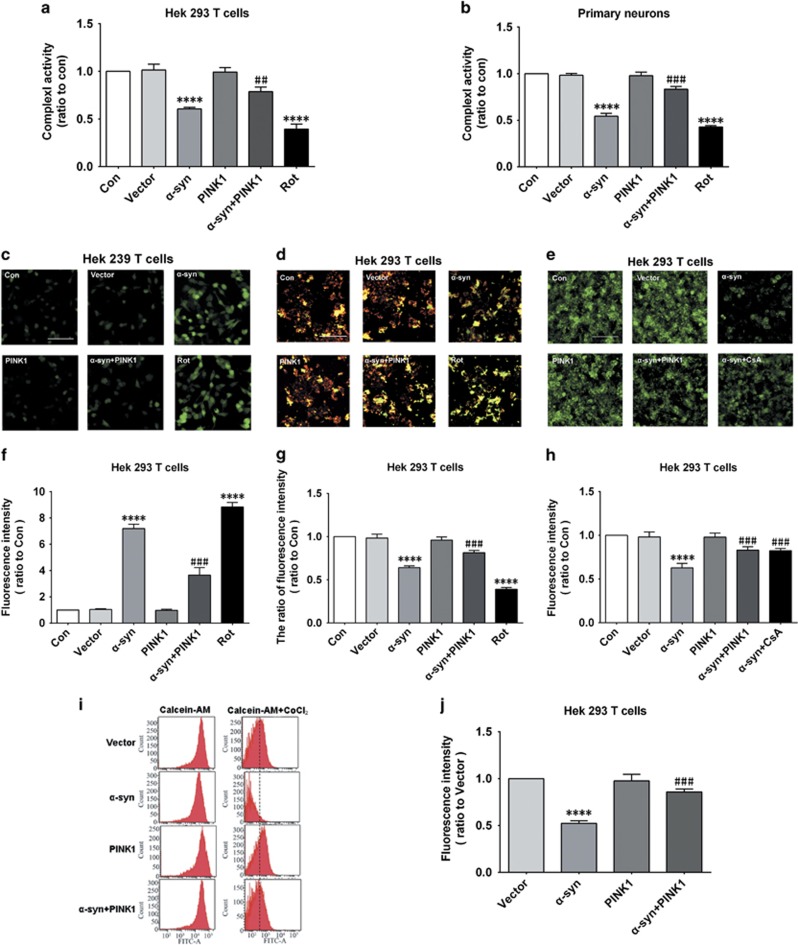
PINK1 protects mitochondria against *α*-syn-induced dysfunction. (**a**,**b**). Mitochondrial complex I activity was detected in HEK293T cells and primary rat cortical neurons. Rotenone (Rot)-treated cells served as a positive control. (**c**). ROS production detected with dichlorofluorescein diacetate in HEK293T cells. Rot-treated cells served as a positive control. (**d**). MMP was detected with JC-1 in HEK293T cells. Rot-treated cells served as a positive control. (**e**) mPTP opening was detected using calcein-AM/CoCl_2_ in HEK293T cells and was assessed by high-content analysis. Cyclosporin A-treated cells served as a positive control. (**f**). Quantitative analysis of fluorescence intensity in dichlorofluorescein diacetate-treated cells. (**g**). Quantitative analysis of fluorescence intensity in JC-1-treated cells. (**h**). Quantitative analysis of fluorescence intensity by high content-analysis in calcein-AM/CoCl_2_-treated cells. (**i**). mPTP opening was detected using calcein-AM/CoCl_2_ in HEK293T cells and was assessed by flow cytometry. (**j**). Quantitative analysis of fluorescence intensity by flow cytometry in calcein-AM/CoCl_2_-treated cells. Data are expressed as mean±S.D. (one-way analysis of variance). ^##^*P*<0.01, ^###^*P*<0.001 *versus α*-syn; ****P<0.0001 *versus* control (Con) (*n*=3)

Given that PINK1 is involved in the maintenance of mitochondrial function, we investigated whether PINK1 could rescue mitochondrial deficits caused by *α*-syn overexpression. We found that PINK1 prevented the decrease in mitochondrial complex I activity induced by *α*-syn overexpression in HEK293T cells and rat primary neurons, rotenone (Rot, 100nM for 24 h), a mitochondrial complex I inhibitor, play as a positive control^18^ (Figures 2a and b). Mitochondrial complex I activity plays an important role in mitochondrial respiratory chain function; its perturbation can lead to the production of reactive oxygen species (ROS).^19^ We found that the increase in ROS induced by *α*-syn overexpression was alleviated by PINK1 (Figures 2c and f). The stability of the mitochondrial membrane, as evidenced by MMP, was assessed using JC-1 dye. *α*-syn overexpression decreased MMP in HEK293T cells, which was abrogated by PINK1 co-expression (Figures 2e and g).

The mitochondrial permeability transition pore (mPTP) is essential for maintaining mitochondrial function; its opening can lead to a decrease in MMP.^20, 21^ We therefore examined the change in mPTP upon *α*-syn with or without PINK1 overexpression using calcein-AM and CoCl_2_. Mitochondrial release of calcein-AM was elevated in cells overexpressing *α*-syn, indicating the opening of the mPTP; however, this was blocked by PINK1, as did the positive control cyclosporine (Cs)A (100nM for 24 h), an inhibitor of mPTP^22^ (Figures 2e). These results indicate that PINK1 prevents the decrease in mitochondrial complex I activity and increase in ROS production induced by *α*-syn overexpression, and stabilizes MMP by inhibiting mPTP opening.

### PINK1 inhibits apoptosis induced by *α*-syn overexpression

**Figure 3:**
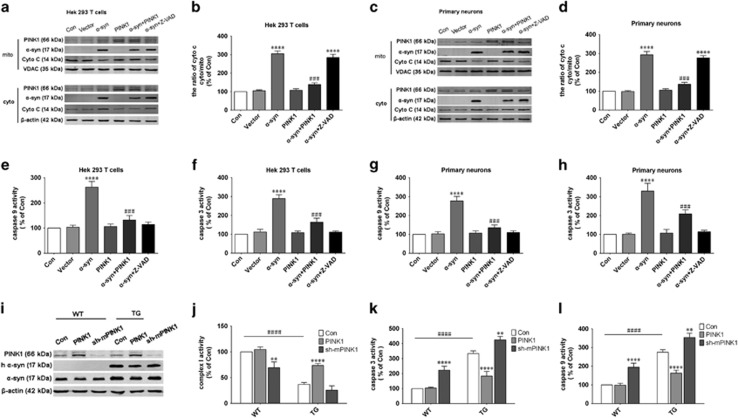
PINK1 inhibits apoptosis induced by *α*-syn overexpressed cells and *α*-syn transgenic mice. (**a**,**c**). Release of Cyto C from mitochondria into the cytoplasm was determined by western blotting in HEK293T cells (**a**) and rat primary cortical neurons (**c**), Z-VAD (50 *μ*M for 24 h) played as a positive control. (**b**,**d**) Quantification of Cyto C in cytoplasm and mitochondria of HEK293T cells (**b**) and rat primary cortical primary neurons (**d**,**e**–**h**). Caspase-3 and -9 activities in HEK293T cells (**e**,**f**) and rat primary cortical primary neurons (**g**,**h**). Data are expressed as mean±S.D. (one-way analysis of variance), ^###^*P*<0.001 *versus α*-syn; ****P<0.0001 *versus* control (*n*=3). (**i**) PINK1 or sh-mPINK1 was injected into the striatum of WT or Thy-1 *α*-syn transgenic mice; after 4 weeks, PINK1 and *α*-syn levels were detected by western blotting. (**j**) Complex I activity in midbrain tissue. (**k**,**l**) Caspase-3 and -9 activity in midbrain tissue. Data are expressed as mean±S.D. (two-way analysis of variance). ^####^*P*<0.0001*versus* control (Con) in the WT group. **P<0.01, ****P<0.0001 *versus* control (Con) (*n*=3)

To determine whether PINK1 protects cells from *α*-syn-induced apoptosis, we separated the mitochondrial and cytoplasmic fractions and compared the levels of cytochrome (Cyto) C. Cells overexpressing *α*-syn had higher levels of Cyto C in mitochondria relative to control cells, which were abolished by PINK1 co-expression (Figures 3a–d). We also found that caspase-3 and -9 activities increased after *α*-syn overexpression, an effect that was reversed by PINK1 (Figures 3e–h). A pan-caspase inhibitor Z-VAD (50 *μ*M for 24 h), as a positive control, abolished the activation of caspase-3 and -9 activities induced by *α*-syn overexpression, while it cannot inhibit Cyto C releasing (Figures 3a–h). These findings indicate that PINK1 counters *α*-syn-induced mitochondrial dysfunction by inhibiting mitochondrial-dependent apoptosis.

### PINK1 restores mitochondrial complex I activity and inhibits apoptosis in *α*-syn transgenic mice

The protective effects of PINK1 in *α*-syn transgenic mice (Tg) were investigated by knocking down mouse PINK1 and overexpressing human PINK1 (Figure 3i), and evaluating mitochondrial complex I activity. PINK1 prevented the decrease in complex I activity resulting from *α*-syn overexpression (Figures 3j) and inhibited the activation of caspase-3 and -9 induced by *α*-syn (Figures 3k and l). These results confirm that PINK1 prevents *α*-syn-induced apoptosis *in vivo*.

### PINK1 prevents *α*-syn localization to mitochondria

**Figure 4:**
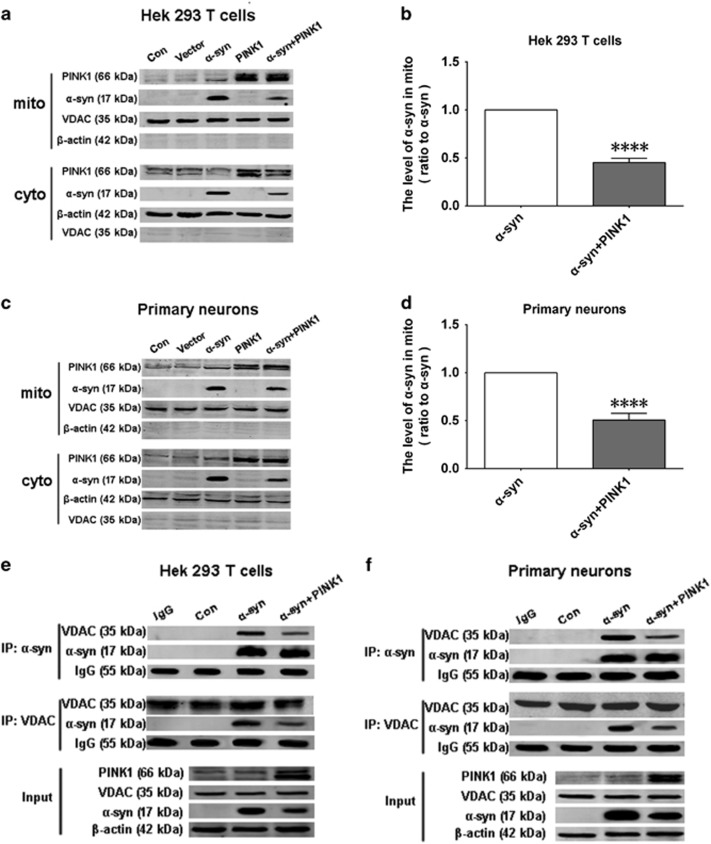
PINK1 prevents localization of *α*-syn to mitochondria. (**a**,**c**) Mitochondria and cytoplasmic fractions were separated, and *α*-syn level was measured to evaluate *α*-syn translocation to mitochondria in HEK293T cells (**a**) and primary rat cortical neurons (**c**). (**b,d**) Quantitative analysis of *α*-syn levels in mitochondrial fractions from HEK293T cells (**b**) and rat primary cortical neurons (**d**–**f**). Interaction of *α*-syn and the mitochondrial membrane protein VDAC, as determined by Co-IP in lysates of HEK293T cells (**e**) and rat primary cortical neurons (**f**). Data are expressed as mean±S.D. (one-way analysis of variance).****P<0.0001 *versus* control (Con) (*n*=3)

Since *α*-syn can associate with mitochondrial membrane and perturb mitochondrial function, we measured the levels of *α*-syn in mitochondrial and cytoplasmic fractions and found that PINK1 overexpression reduced the mitochondrial *α*-syn level in HEK293T cells and rat primary neurons (Figures 4a–d). We also examined the interaction of *α*-syn and voltage-dependent anion channel (VDAC), a mitochondrial outer membrane protein. Overexpressed *α*-syn interacted with VDAC, but this association was reduced in the presence of PINK1 (Figures 4e and f), indicating that PINK1 blocks mitochondrial localization of *α*-syn.

### PINK1 interacts with *α*-syn in the cytoplasm via the kinase domain

**Figure 5:**
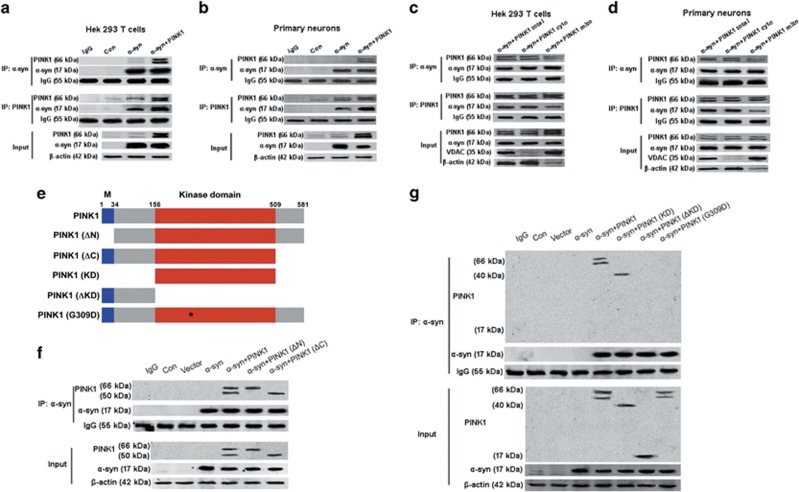
PINK1 interacts with *α*-syn via its kinase domain mainly in the cytoplasm. (**a**,**b**) Interaction of PINK1 and *α*-syn detected by Co-IP in HEK293T cells (**a**) and rat primary cortical neurons (**b**–**d**). Interaction of PINK1 and *α*-synin mitochondrial and cytoplasmic fractions of HEK293T cells (**c**) and rat primary cortical neurons (**d**). (**e**) A schematic depiction of PINK1 constructs. M: mitochondrial targeting sequence. (**f**) HEK293T cells were transfected with *α*-syn and full-length PINK1 or PINK1 N-terminal deletion (PINK1 (ΔN) or C-terminal deletion [PINK1 (ΔN)] PINK1 interaction with *α*-syn was detected by co-IP. (**g**) HEK293T cells were transfected with *α*-syn and full-length PINK1 or the PINK1 kinase domain [PINK1 (KD)], kinase domain deletion mutant [PINK1 (ΔKD)] or PINK1 kinase domain inactivation mutant [PINK1 (G309D)]. PINK1 interaction with *α*-syn was detected by co-IP (*n*=3)

**Figure 6:**
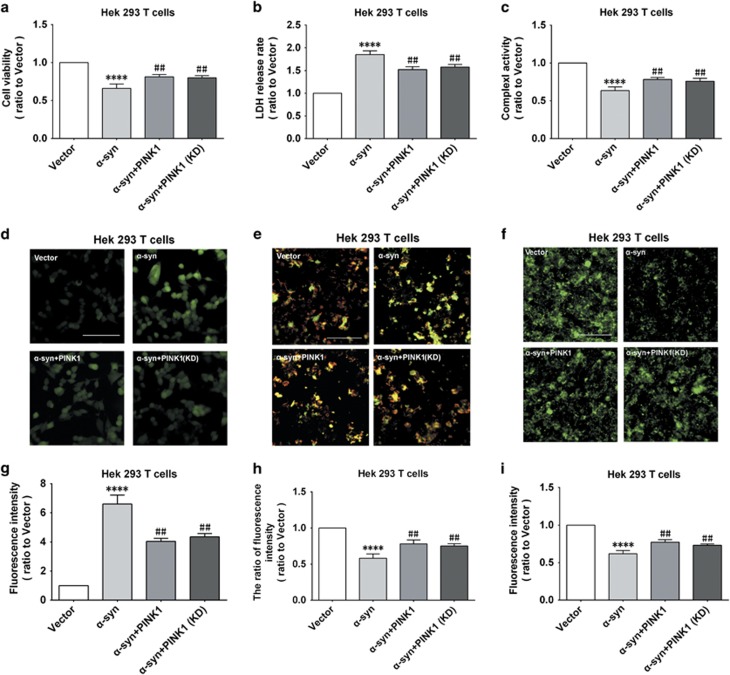
PINK1 kinase domain rescues *α*-syn-induced reductions in viability and mitochondrial function in HEK293T cells. (**a,b**) Cell viability and cytotoxicity were evaluated with the MTT and LDH assays, respectively. (**c**) Mitochondrial complex I activity. (**d**) ROS production as detected by dichlorofluorescein diacetate. (**e**) MMP as detected by JC-1 staining. (**f**) mPTP as detected by calcein-AM/CoCl_2_ staining. (**g**–**i**) Quantitative analysis of fluorescence intensity in cells treated with dichlorofluorescein diacetate (**g**), JC-1 (**h**), and calcein-AM/CoCl_2_ (**i**). Data are expressed as mean±S.D. (one-way analysis of variance). ^##^*P*<0.01, ^###^*P*<0.001 *versus α*-syn; ****P<0.0001 *versus* control (Con) (*n*=3)

To clarify the mechanism by which PINK1 prevents mitochondrial localization of *α*-syn, we examined whether the two proteins directly interact in co-immunoprecipitation (Co-IP) experiments. *α*-syn immunoprecipitated with PINK1 from HEK293T cells and rat primary neurons (Figures 5a and b), but this interaction was mainly in the cytoplasm fraction, but not in the mitochondrial fraction (Figures 5c and d). We investigated the domain of PINK1 that was responsible for this interaction by transfecting HEK293T cells with several PINK1 constructs, including PINK1 (ΔN), PINK1 (ΔC), PINK1 kinase domain (KD), PINK1 (ΔKD) and PINK1 G309D, which is a kinase domain inactive mutation^23^ (Figure 5e). Co-IP experiments revealed that N- or C-terminal deletion had no effect on the interaction between *α*-syn and PINK1 (Figure 5f). Interestingly, our results showed that *α*-syn immunoprecipitated with PINK1 (KD) but not PINK1 (ΔKD); moreover, the G309D mutation of PINK1 also prevented its interaction with *α*-syn (Figure 5g). Overexpressing the PINK1 (KD) rescued the decrease in cell viability resulting from *α*-syn overexpression (Figures 6a and b) and enhanced the mitochondrial function by increasing complex I activity, reducing ROS production and stabilizing MMP by blocking mPTP opening (Figures 6c–i). These results indicate that PINK1 interact with *α*-syn via its kinase domain in the cytoplasm.

### PINK1 induces *α*-syn degradation via autophagy

**Figure 7:**
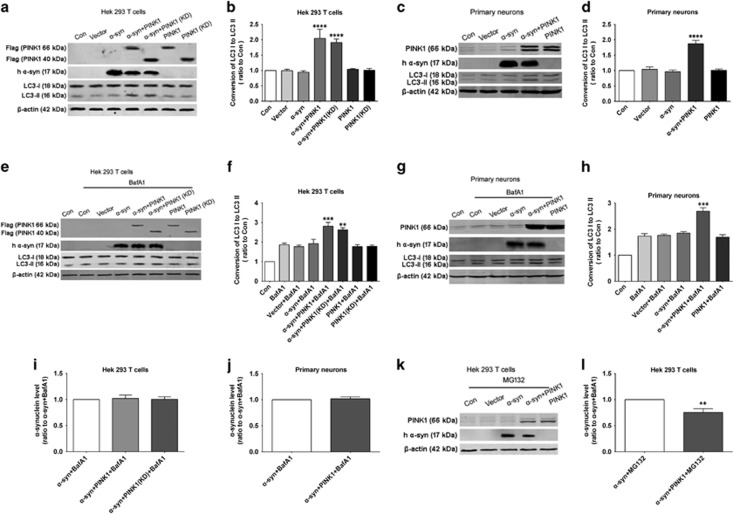
PINK1 promotes *α*-syn degradation via autophagy. (**a**) HEK293T cells were transfected with *α*-syn, PINK1 and PINK1 kinase domain [PINK1 (KD)], LC3-I and -II expression was evaluated by western blotting. (**b**) Quantification of LC3-I/II conversion in HEK293T cells. (**c**) Primary neurons were infected with *α*-syn and PINK1, LC3-I and -II expression was evaluated by western blotting. (**d**) Quantification of LC3-II/I ratio in primary neurons. (**e**) HEK293T cells were transfected with *α*-syn, PINK1 and PINK1 kinase domain [PINK1 (KD)] in the present of autophagy flux inhibitor Bafilomycin A1 (BafA1), LC3-I and -II expression was evaluated by western blotting. (**f**) Quantification of LC3-I/II conversion in HEK293T cells. (**g**). Primary neurons were infected with *α*-syn and PINK1 in the present of autophagy flux inhibitor Bafilomycin A1 (BafA1), LC3-I and -II expression was evaluated by western blotting. (**h**). Quantification of LC3-II/I ratio in primary neurons. (**i**,**j**) Quantification of *α*-syn level in HEK293T cells or primary neurons in the present of BafA1. (**k**) HEK293T cells were transfected with *α*-syn and PINK1 in the present of proteasome inhibitor MG132, *α*-syn level was evaluated by western blotting. (**l**) Quantification of *α*-syn level in HEK293T cells in the present of MG132. Data are expressed as mean±S.D. (one-way analysis of variance). ****P<0.0001 *versus* control (Con), **P<0.01, ***P<0.001 *versus* BafA1 or *α*-syn +MG132. (*n*=3). h *α*-syn, human *α*-syn

Our observation that cytoplasmic *α*-syn levels decreased upon co-expression with PINK1 suggested that *α*-syn may be targeted for degradation. It was previously found that *α*-syn is degraded via the ubiquitin proteasome system or by autophagy, so we evaluated autophagy induction by measuring microtubule-associated protein light chain (LC)3-II level. The results showed that LC3-II was markedly increased upon co-expression of *α*-syn and PINK1 or its kinase domain in HEK293T cells or primary neurons (Figures 7a–d.). To further evaluate autophagy induction, we inhibited autophagic flux by using Bafilomycin A1 (BafA1, 100 nM for 6 h), similarly, PINK1 or PINK1 (KD) co-overexpressed with *α*-syn increased LC3-II level obviously, confirming the induction of autophagy after *α*-syn and PINK1 co-overexpression (Figures 7e and h). Interestingly, we found *α*-syn level decreased in co-overexpressed group (Figures 7a), while these decreasing were abolished in the present of BafA1, indicating that *α*-syn degraded by autophagy (Figures 7i and j). Although ubiquitin proteasome system is another pathway related to *α*-syn degradation, while we found co-expression of *α*-syn and PINK1 also decreased *α*-syn level in the present of MG132 (5 *μ*M for 24 h), an inhibitor of proteasome, indicating that *α*-syn was not degraded by ubiquitin proteasome system (Figures 7k and l). These results suggested that PINK1 promotes *α*-syn degradation in the cytoplasm via autophagy.

### *α*-syn increases endogenous PINK1 expression

Our finding that *α*-syn overexpression increased endogenous PINK1 levels in HEK293T cells and rat primary neurons was confirmed in the brain tissue of *α*-syn transgenic mice by real-time (RT-) polymerase chain reaction (PCR) analysis of *PINK1* expression, which was upregulated by *α*-syn overexpression Supplemental Figure 1). Thus, endogenous PINK1 may exert a protective effect against *α*-syn-induced toxicity *in vivo*.

## Discussion

**Figure 8:**
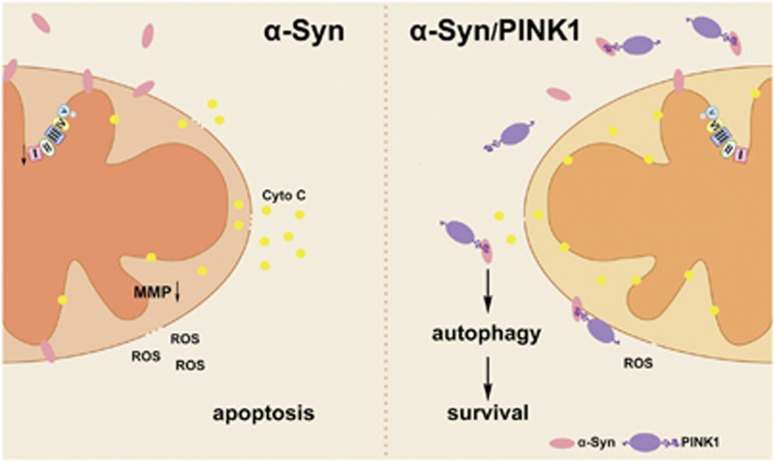
PINK1 exerts protective effects against *α*-syn-induced cytotoxicity. *α*-Syn causes mitochondrial deficits and cell death by localizing to mitochondria and activating apoptosis. PINK1 interacts with excess *α*-syn in the cytoplasm to prevent its localization to mitochondria and thereby inhibit apoptosis caused by mitochondrial damage. Upon its interaction with PINK1, excess *α*-syn is eliminated through autophagy

In this study, we confirmed that PINK1 interacts with *α*-syn and induces its degradation via autophagy. In addition, PINK1 suppressed *α*-syn overexpression-induced mitochondrial dysfunction and apoptosis (Figure 8).

It was previously reported that *α*-syn overexpression decreased cell viability and caused behavioral deficits in PD models, effects that were abrogated by PINK1.^13, 14, 15^ Many studies have reported that *α*-syn localizes to and impairs mitochondrial function.^7, 24, 25^ In the present study, we found that PINK1 mainly interacted with *α*-syn in the cytoplasm, preventing it from associating with mitochondria. The PINK1–*α*-syn interaction resulted in *α*-syn degradation by autophagy.

PINK1 has been shown to be involved in mitophagy initiation in the presence or absence of Parkin, another PD-related protein.^26, 27^ Autophagy differs from mitophagy; indeed, we found that PINK1 interacted with *α*-syn and recruited ubiquitin in the cytoplasm and not in the mitochondrial fraction. This suggests that autophagy induced by the PINK1–*α*-syn complex was only responsible for eliminating excessive *α*-syn so as to prevent it from impairing mitochondrial function, which can be described as macroautophagy rather than mitophagy.

PINK1 overexpression alleviated *α*-syn-induced mitochondrial deficits and inhibited apoptosis. We also found that *α*-syn overexpression increased PINK1 mRNA and protein levels. These findings suggest that endogenous PINK1 has a protective effect against *α*-syn-induced cytotoxicity. Consistent with our results, another study found that PINK1 transcription was upregulated in Thy-1 *α*-syn transgenic mice as compared to wild-type mice.^27^ Moreover, PINK1 protein level was increased in *α*-syn transgenic mice treated with Rot, a pesticide that is used for animal models of PD, indicating that *α*-syn overexpression may stimulate endogenous PINK1 as a compensatory response to cellular stress.^28^

In this study, we showed that deletion, G309D mutation or inactivating mutation of the PINK1 kinase domain prevented the interaction with *α*-syn. As a Ser/Thr kinase, PINK1 may phosphorylate *α*-syn, although a phosphorylation site has yet to be identified. Recent studies found that PINK1 was phosphorylated ubiquitin at Ser65,^29, 30, 31^ which was associated with Parkin activation. Phosphorylated ubiquitin modulates two autophagy receptors—optineurin and nuclear domain 10 protein 52 (NDP52)—which are recruited to mitochondria and induce mitophagy.^26, 27^ We found here that the PINK1–*α*-syn complex recruited ubiquitin in the cytoplasm and induced autophagy. Additional studies are needed in order to identify the autophagy receptor involved in these events.

In conclusion, our results demonstrate that PINK1 interacts with *α*-syn via its kinase domain and induces autophagy in the cytoplasm, thereby preventing *α*-syn from localizing to mitochondria and inducing apoptosis. These results highlight a novel mechanism underlying the protective effect of PINK1 against *α*-syn-induced neurodegeneration as well as a possible therapeutic target for PD treatment.

## Materials and methods

### Plasmids and lentivirus

WT human *α*-syn cDNA (GenBank accession no. NM 000345) was subcloned into pLNCX2 or pCMV-Myc vector (Clontech, Mountain View, CA, USA). Insert orientation and sequence were verified by DNA sequencing. Plasmids encoding pcDNA3.1-3 × Flag-hPINK1WT (WT PINK1; kindly provide by Changan Jiang, Sichuan University), pcDNA3.1-3 × Flag-hPINK1 (ΔN,PINK1 without the mitochondrial targeting signal), pcDNA3.1-3 × Flag-hPINK1 (ΔC, PINK1 without the Cterminus), pcDNA3.1-3 × Flag-hPINK1 G309D (G309D mutant of PINK1 lacking kinase activity), pcDNA3.1-3 × Flag-hPINK1 (ΔKD, PINK1 lacking the kinase domain and Cterminus), pcDNA3.1-3 × Flag-hPINK1 (KD, kinase domain of PINK1), lentivirus (LV)-WT-*α*-syn and LV-WT-PINK1 were generated by Genechem (Shanghai,China).

### Cell culture and infection

HEK293T cells were cultured in Dulbecco’s Modified Eagle’s Medium supplemented with10% heat-inactivated fetal bovine serum (10099-141; Gibco, Grand Island, NY, USA). At 80% confluence, HEK293T cells were transfected with the appropriate plasmid using Lipofectamine 2000 (11668019, Invitrogen, Carlsbad, CA, USA) according to the manufacturer’s instructions. At specific times after transfection, cells were harvested for experiments.

### Primary cortical neurons

Experiments involving animals were approved by the Institutional Animal Care and Use Committee of Capital Medical University of Science and Technology (Beijing, China; approval no. SCXK-2011-004) and were carried out in accordance with the National Institutes of Health Guide for the Care and Use of Laboratory Animals (Publication no. 80-23). Primary cortical neurons cultured as previous described.^32^ Briefly, surgeries were performed under sodium pentobarbital anesthesia. Primary cortical neurons were prepared from Sprague-Dawley rats or C57BL mouse E14.5–E15.5 embryos and cultured in 3.5cm dishes (1.4 × 10^6^ cells/dish) on cover slips coated with 100 *μ*g/ml poly-L-lysine in neurobasal medium (21103–049; Invitrogen) supplemented with basic fibroblast growth factor (10 ng/ml), nerve growth factor (10 ng/ml), L-glutamine (0.5 mM) and B27 supplement. After 7 days, primary neurons were infected with LV gene transfer vectors.

### Transgenic mice

Male Thy-1 *α*-syn transgenic mice (18–22 g) were purchased from the Jackson Laboratory (Bar Harbor, ME, USA) and maintained on a C57BL/6N background. Transgenic mice and WT littermates were housed under a 12:12-h light/dark cycle at 20 °C–25 °C with free access to food and water.

### MTT and LDH assays

Cell viability was determined with the MTT assay. Briefly, cells were seeded in 96-well microplates (1 × 10^4^ cells/well) and transfected or infected with appropriate vectors. After 24 h, the medium was replaced with MTT (Promega, Madison, WI, USA) at a final concentration of 0.5 mg/ml and incubated for 4h. Cells were washed twice with phosphate-buffered saline (PBS); formazan crystals were dissolved in 100 *μ*l dimethyl sulfoxide, and absorbance was read at 490 nm on a microplate reader (PerkinElmer, Waltham, MA, USA).

The LDH assay was performed using a kit (Roche Diagnostics, Mannheim, Germany) according to the manufacturer’s instructions. A 100 *μ*l aliquot of each supernatant was used to measure LDH release, with 100 *μ*l of preservation solution used as a blank to correct the optical density reading at 490 nm. Each concentration was tested in triplicate, and the half-maximal effective concentration was averaged from five experiments.

### Complex I activity assay

Cells were homogenized mechanically in 10 mM Tris-HCl (pH7.2) containing 225 mM mannitol, 75 mM saccharose and 0.1 mM EDTA, and then centrifuged at 600 × *g* and 4 °C for 20 min to obtain the post-nuclear supernatant. Complex I activity was determined by measuring nicotinamide adenine dinucleotide (NADH) oxidation at 37 °C over 3 min by spectrophotometry (340 nm). The assay medium contained 40 *μ*g of protein from the post-nuclear supernatant dissolved in 1 ml of 25 mM phosphate buffer (pH 7.5), 2.5 mg/ml bovine serum albumin, 100 *μ*M decylubiquinone and 200 *μ*M NADH. The complex I inhibitor Rot (2 *μ*M) was used to determine the fraction of NADH that was oxidized independently of complex I (blank values).

### Measurement of intracellular ROS levels

To evaluate intracellular ROS production, cells were incubated with the fluorescent probe dichlorofluorescein diacetate (1 *μ*M; Sigma-Aldrich, St. Louis, MO, USA) at 37 °C in the dark for 30 min, then collected, washed with 0.01 M PBS, centrifuged and resuspended in 400 *μ*l PBS. The green fluorescence intensity (516 nm) was quantified by high-content analysis with excitation and emission wavelengths of 488 and 530 nm, respectively.

### JC-1 staining for determination of MMP

MMP was measured using JC-1 (T4069; Sigma-Aldrich), a dual-emission membrane potential-sensitive probe that exists as a green fluorescent monomer at low MMP, but has red/orange fluorescence in the J-aggregate form at higher MMP. JC-1 (1.3 *μ*g/ml) was added to cells cultured in 24-well plates after washing twice with PBS for 30 min at 37 °C. The change in fluorescence at 488/530 nm (green) and 549/595 nm (red) was monitored by high-content screening, and the ratio of green/red fluorescence intensity was determined.

### Calcein-AM

mPTP activation in cells grown in a 24-well plate was determined by monitoring calcein-AM fluorescence using the Mito Probe Transition Pore Assay kit (Invitrogen; M34153) according to the recommended protocol. Briefly, cells were incubated with calcein-AM and CoCl_2_ with or without ionomycin in Hank’s balanced salt solution (HBSS)/Ca^2+^ at 3 7°C for 15 min while protected from light. After two washes with HBSS/Ca^2+^, calcein-AM fluorescence was detected by flow cytometry and high-content screening at 488/530 nm.

### Co-IP

Cell extracts (100 *μ*g) from all pretreated protein groups were precleared with Protein-G agarose (Pierce, Rockford, IL, USA) and then incubated with anti-*α*-syn (1:1000; BD Biosciences, Franklin Lakes, NJ, USA) and rabbit anti-PINK1 (1:1000; Novus Biologicals, Littleton, CO, USA) antibodies at 4 °C overnight with constant rotation. Protein G-sepharose beads (30 *μ*l/tube) were washed three times for 15 min in IP buffer composed of 10 mM Tris-Cl (pH 7.5),150 mM NaCl, 2 mM EDTA and 0.5% Triton 100, then incubated with protein/antibody mixture at 4 °C for 6 h with constant rotation. The precipitant was collected by centrifugation at 10 000 × *g* for 1 min and washed three times with IP buffer to remove non-specifically bound proteins. The washed beads were resuspended in sodium dodecyl sulfate polyacrylamide gel electrophoresis (SDS-PAGE) loading buffer (30 *μ*l/tube) and heated at 95 °C for 5 min, and then removed by centrifugation at 10 000 × *g* for 1 min. The supernatant was analyzed by SDS-PAGE and western blotting.

### Mitochondria and cytoplasm isolation

Mitochondria were isolated from transfected HEK293T cells or primary neurons using the Mitochondria/Cytosol Fractionation kit (Applygen Technologies, Beijing, China; C1260) as previously reported.^33^

### Western blotting

After transfection or infection, cells were washed with ice-cold PBS and lysed in radio immunoprecipitation buffer composed of 50 mM Tris-Cl (pH 7.4), 150 mM NaCl, 1% Nonidet P-40, 0.1% SDS, and a phosphate and protease inhibitor cocktail (Roche Diagnostics; 04693132001). Homogenates were centrifuged at 12 000 × *g* for 20 min and the supernatant was collected for analysis. The protein concentration was determined using a bicinchoninic acid protein assay kit (Pierce; 23227) according to the manufacturer’s instructions. A total of 20 *μ*g of protein was resolved on 10% SDS polyacrylamide gels and transferred to a polyvinylidene difluoridemembrane that was blocked with 10% skim milk for 1 h and then incubated with the following primary antibodies: rabbit anti-*β*-actin (1:1000; Sigma), rabbit anti-Flag (1:1000; Sigma), mouse anti-Myc (1:1000; Sigma), mouse anti-*α*-syn (1:1000; BD Biosciences), mouse anti-human *α*-syn (3D5) gift from Prof. Shun Yu at Xuanwu Hospital of Capital Medical University, Beijing, China,^34^ rabbit anti-PINK1 (1:1000; Novus Biologicals), goat anti-VDAC (1:1000, Santa Cruz Biotechnology, Santa Cruz, CA, USA), rabbit anti-cytochrome C(1:1000; Sigma) and rabbit anti-LC3 (1:1000; Novus Biologicals). Horseradish peroxidase-conjugated secondary antibodies (1:10 000) were purchased from Santa Cruz Biotechnology. *β*-actin and VDAC were used as loading controls. Immunoreactivity was visualized with super-enhanced chemiluminescence detection reagent (Applygen Technologies, Beijing, China; P1020) using a Gel-Doc 2000 imaging system (Bio-Rad).

### Caspase-3 and -9 activity assays

Caspase-3 and -9 activities were measured with Caspase-3 and -9 colorimetric assay kits (Applygen; C1113 and C1119, respectively). The assay is based on spectrophotometric detection of the chromophore p-nitroanilide (pNA) after its cleavage from the labeled substrate LEHD-pNA. After transfection for 48h, the cells were centrifuged at 1000 × *g* for 5 min. A 60 *μ*l volume of cell lysis buffer was added and the mixture was vortexed and incubated on ice for 10 min, followed by vortexing and centrifugation at 12 000 × *g* for 10 min at 4 °C. Proteins were transferred to another 1.5 ml centrifuge tube and the protein concentration was determined using a Bradford assay kit (GenMed Scientifics, Shanghai, China; GMS 30030.1). A 50 *μ*l volume of protein was added to the 96-well plate, and 45 *μ*l reaction buffer was added to each sample; 5 *μ*l of 2 mM LEHD-pNA (100 *μ*M final concentration) were then added, and the plate was sealed with sealing film and incubated at 37 °C for 2 h. The absorbance of samples was read at 405 nm with a spectrophotometer. One unit was defined as the amount of enzyme that would cleave 1.0 nM of the colorimetric pNA-substrate per hour at 37 °C under saturated substrate concentrations.

### RT-PCR

Total RNA from HEK293T cells was prepared using the RNeasy kit (Qiagen, Valencia, CA, USA) and then reverse-transcribed into cDNA using the Transcriptor High Fidelity cDNA Synthesis kit (Roche Diagnostics) according to the manufacturer’s instructions. Quantitative RT-PCR was performed with SYBR GreenER (Invitrogen) on a thermal cycler (Bio-Rad). The following forward and reverse primers were used: mouse *β*-actin, 5′-ACC TTC TAC AAT GAG CTG CG-3′ and 5′-GTG GAT GGCC TAC GTA CAT GC-3′ mouse PINK1,AGA 5′-AAA CCA AGC GCG TGT CT-3′ and 5′-GGA AGC CCT GCC AGC AT-3′ and human *α*-syn, 5′-CCA GTT GGG CAA GAA TGA AGA-3′ and 5′-AAG CCT CAT TGT CAG GAT CCA-3′.

### Statistical analysis

Mean differences were evaluated by analysis of variance followed by a Bonferroni *post hoc* test using Prismv.6.0 software (GraphPad Inc., La Jolla, CA, USA). Data are expressed as mean±S.D., and a *P*-value<0.05 was considered statistically significant.

## Publisher’s Note:

Springer Nature remains neutral with regard to jurisdictional claims in published maps and institutional affiliations.
