# Supplementary figures and images for: Pink1 interacts with α-synuclein and abrogates α-synuclein-induced neurotoxicity by activating autophagy
Source: Cell Death Dis. 2017 Sep 21;8(9):e3056–. doi: 10.1038/cddis.2017.427 (PMC5636973; doi:10.1038/cddis.2017.427)

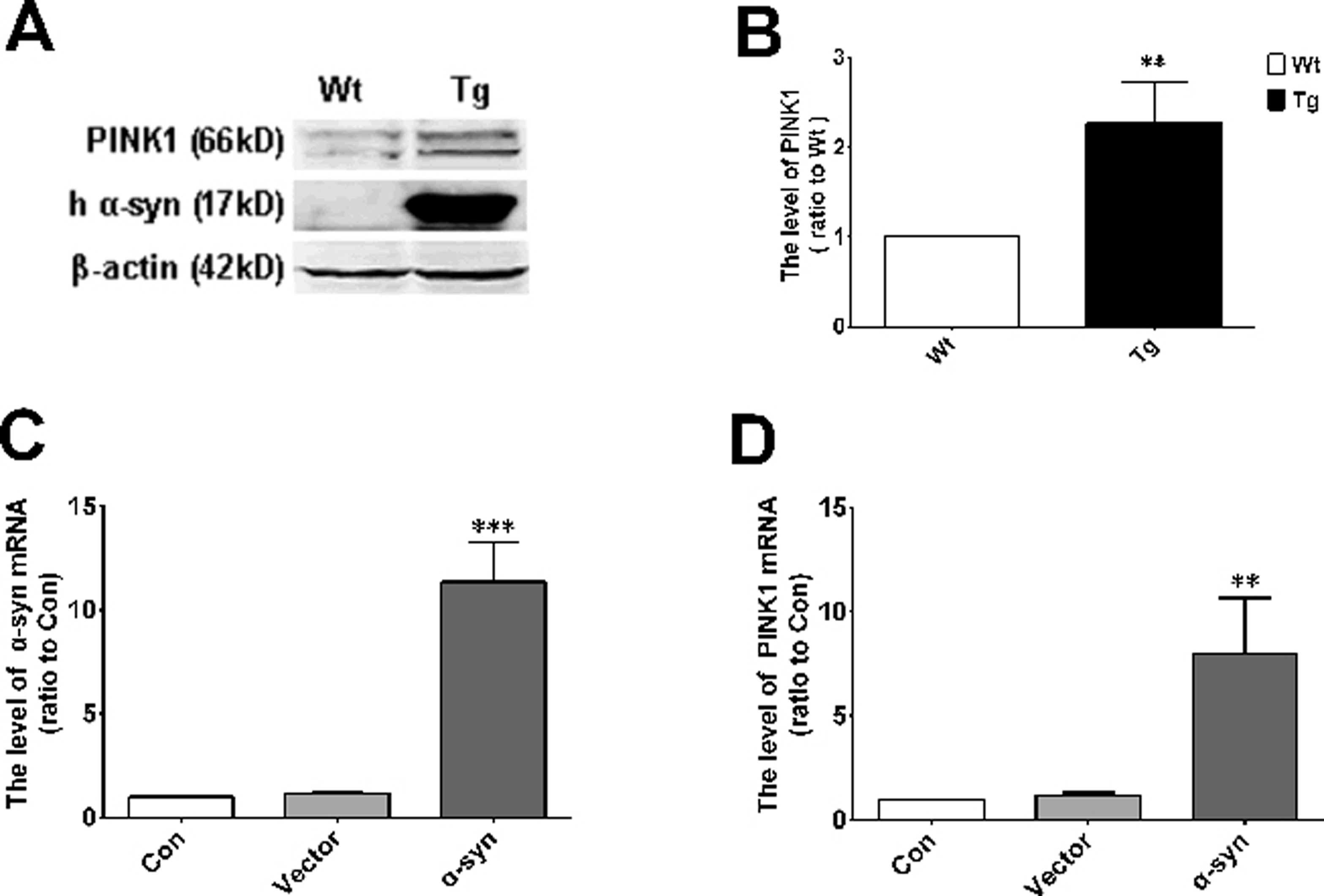

Supplement: Supplementary Figure [file cddis2017427x1.tif]
