# Supplementary material for: Pink1 interacts with α-synuclein and abrogates α-synuclein-induced neurotoxicity by activating autophagy
Source: Cell Death Dis. 2017 Sep 21;8(9):e3056–. doi: 10.1038/cddis.2017.427 (PMC5636973; doi:10.1038/cddis.2017.427)
Supplement: Supplementary Figure Legends [file cddis2017427x2.docx]

**Supplementary Figure Legend**

**S1.** α-Syn overexpression induces endogenous PINK1 expression. **A.**PINK1 levels detected by western blotting in brain tissue of α-syn transgenic and WT mice. **B.** Quantification of PINK1 levels. **C, D.** HEK293T cells were transfected with α-syn; after 24 h, α-syn and PINK1 levels were detected by RT-PCR. Data are expressed as mean ± SD (one-way analysis of variance). **P<0.01, ***P<0.001 vs. WT or control (Con) (n=3).
